# Cholesteræmia

**Published:** 1873-12

**Authors:** 


### Our Exchanges.

*THE MEDICAL RECORD—December*, 1873:

Cholesteræmia.—A paper was read before the New York Academy of Medicine, November 6th, by Prof. Austin Flint, Jr., upon the subject of "Cholesteræmia." The author of the paper gave an interesting and somewhat exhaustive *resume* of the experiments which he had performed with the view of determining the nature, origin and termination of cholesterine in the human body, the results of which experiments he brought prominently before the profession in the year 1862. Although the views presented by the author of the paper in 1862 have passed comparatively unnoticed in this country and England up to the present time, they have received some attention in France and Germany, and within a few years some experiments have been performed in Germany which have led the author to believe that his own experiments possessed some scientific importance. "It is well known," says the author of the paper, "that there are two classes of diseases widely differing in their gravity. In the first place there is the ordinary attack of jaundice, which is not very severe, not very dangerous, is relieved by some simple remedy, or relieves itself after a very few days. But there are other cases of jaundice, in which the icterus is not more intense, may be even less intense, than in these moderate cases, but which are characterized from the very beginning by very grave symptoms, referable to the nervous system, and almost invariably terminate in death.

In occasional cases of cirrhosis of the liver, there are manifested grave disturbances, referable to the nervous system, with more than an ordinarily severe sequence from the pathological lesion existing in the organ.

An effort has been made to explain the pathology of such striking cases by supposing that some of the constituents of the bile have been retained in the blood, which should have been eliminated; but physiological experiment has never verified the supposition. The reason why the biliary salts have not been found in the blood is because they are not secreted. "The distinction between secretion and excretion is almost absolute. The elements of secretion do not pre-exist in the blood, but are manufactured in the substance of glands out of materials furnished by the blood; that their flow, as a rule, is when there is necessity for their function; that when the function of the glands is interfered with, the materials do not accumulate in the blood. The elements of excretion always pre-exist in the blood; they are never manufactured by special organs, but are the result of the general processes of dis-assimilation; they are not manufactured by glands, but separated from the blood by glands, and when the function of excreting organs is interfered with, an accumulation of the elements occurs in the blood, and produces a toxæmic effect."

The Professor then gave a somewhat extended but interesting account of the experiments which he had performed to demonstrate that cholesterine is always found in the blood, is always found in the nervous tissue, and is always found in the bile.

It has been found by his experiments that the blood gains something over twenty-three per cent. of cholesterine while passing through the brain, and loses cholesterine in very near the same proportion while passing through the liver.

It has also been found that, in cases of hemiplegia of some standing, the blood taken from the affected side contained *no* cholesterine, while upon the unaffected side it was found as in normal blood. These experiments proved that cholesterine is formed in nervous tissue, is eliminated by the liver, and is therefore placed among the excrementitious substances, and its disappearance from the body is under the form of stercorine.

"If it be true," says the Professor, "that it is an excrementitious substance, we should expect that if the liver becomes disorganized, so that it cannot be eliminated, the term cholesteræmia would be justified in science."

The result of examination in a grave case of jaundice, accompanied by serious nervous disturbances, such as stupor, coma, and convulsions, showed extensive structural disease of the liver, and the quantity of cholesterine increased to three times the quantity found in the blood in a case of simple jaundice.

Over 50 per cent. above the average amount of cholesterine has been found in the blood of a patient who died of cirrhosis of the liver, attended by grave nervous disturbances. Finally, the views of the author of the paper are summed up in the following:

"Cholesterine is an excrementitious substance, produced chiefly by dis-assimilation or physiological wear of brain and nervous tissue, is taken up by the blood and carried to a special organ for its elimination, and that organ is the liver. In the liver it is separated from the blood, and finds its way into the bile discharged into the small intestines, and there changed into stercorine, which is discharged from the body at about the rate of ten grains each twenty-four hours. In certain cases of disease of the liver, accompanied by grave nervous symptoms, there is an accumulation of the cholesterine in the blood, giving rise to the condition which has been called cholesteræmia."

The present paper was called out by the report of some experiments which have lately been performed by an eminent German Professor in Leipsic, by injecting cholesterine into the circulation of animals. The results which have been produced will warrant the conclusion, apparently satisfactory at least, that those symptoms which accompany severe jaundice, and are not unfrequently seen in connection with many diseases of the liver, are the manifestations produced by an abnormal accumulation of cholesterine in the blood, which conditions have been improperly recognized under names such as cholæmia and others.

Dr. Barker made the following remarks and suggestions upon the paper read by Prof. Flint: I wish to call attention to certain practical bearings the discovery possesses. There are certain conditions which every man is familiar with, and which are characterized by a well-known mental sluggishness and general torpor of the system, that yield at once to what is termed a free bilious evacuation from the bowels. Bilious attacks, bilious headaches, etc., are the terms commonly applied to such conditions. Now it seems to me the paper upon cholesteræmia proved that through the medium of the liver, there is eliminated from the system an effete *debris* of nerve-tissue, and that the failure in elimination of this material gives rise to those phenomena called "biliousness;" and by the action of proper remedies the effects of this retained element are caused to pass away. One of my patients, an eminent lawyer, was in the habit, for many years, when he was called upon to make a strong mental effort, of taking ten grains of blue-pill, and following it with a bottle of congress water, a few hours before entering upon his labor.

Physiologists have been long accustomed to recognize a certain form of disease, called cholæmia, by the presence of bile in the blood. The culmination of the disease consists in three classes of phenomena: First, those which are referred to the nervous system, such as delirium, convulsions, and sometimes coma; second, those which are referable to the nutritive system, resulting in typhoid symptoms; and, third, a class of phenomena referable especially to the circulatory system, the mucous membranes, and the kidneys, resulting in hemorrhages from the mucous membranes, etc., etc. There are also cases of so-called melæna, which are attended with great disturbance of the nervous system; and the thought has been brought to my mind many times, may not some of the phenomena which have heretofore been classed under the head of bile-poisoning be due to the presence of cholesterine in the blood?

In my own special department I have seen a few cases in which the symptomatic phenomena finally culminated in convulsions, dependent upon the condition of the liver, as I believed, and not upon the condition of the kidneys.

From my experience in connection with some of these cases I am convinced that there is a certain class of cases which are dependent upon cholesteræmia rather than upon uræmia, in which convulsions were developed; and in accordance with that belief I have directed my treatment towards the liver, and have witnessed the most gratifying results.

I simply throw out these hints, which are of a practical nature, and may be of service in the consideration of the paper to which we have listened.

